# The sequence of disease-modifying therapies in relapsing multiple sclerosis: safety and immunologic considerations

**DOI:** 10.1007/s00415-017-8594-9

**Published:** 2017-09-06

**Authors:** Gabriel Pardo, David E. Jones

**Affiliations:** 1 OMRF Multiple Sclerosis Center of Excellence, Oklahoma Medical Research Foundation, 820 NE 15th Street, Oklahoma City, OK 73104 USA; 2 Department of Neurology, University of Virginia School of Medicine, PO Box 800394, Charlottesville, VA 22908 USA

**Keywords:** Multiple sclerosis, Therapeutic drug monitoring, Selection for treatment, Re-treatment, Treatment effectiveness

## Abstract

The treatment landscape for relapsing forms of multiple sclerosis (RMS) has expanded considerably over the last 10 years with the approval of multiple new disease-modifying therapies (DMTs), and others in late-stage clinical development. All DMTs for RMS are believed to reduce central nervous system immune-mediated inflammatory processes, which translate into demonstrable improvement in clinical and radiologic outcomes. However, some DMTs are associated with long-lasting effects on the immune system and/or serious adverse events, both of which may complicate the use of subsequent therapies. When customizing a treatment program, a benefit–risk assessment must consider multiple factors, including the efficacy of the DMT to reduce disease activity, the short- and long-term safety and immunologic profiles of each DMT, the criteria used to define switching treatment, and the risk tolerance of each patient. A comprehensive benefit–risk assessment can only be achieved by evaluating the immunologic, safety, and efficacy data for DMTs in the controlled clinical trial environment and the postmarketing clinical practice setting. This review is intended to help neurologists make informed decisions when treating RMS by summarizing the known data for each DMT and raising awareness of the multiple considerations involved in treating people with RMS throughout the entire course of their disease.

## Introduction

The unpredictable nature of multiple sclerosis (MS) clinical manifestations within and between patients with apparently similar characteristics is brought about by a complex and dynamic pathophysiology involving inflammatory-based mechanisms of demyelination and axon loss [1–3]. A range of genetic [4, 5], immunopathologic [6–8], and environmental/epigenetic [9] factors drive the tremendous variability in the type, frequency, and severity of signs and symptoms that may present during the course of MS [10, 11].

Despite the heterogeneity in MS disease course, selecting an appropriate therapy for relapsing forms of MS (RMS) before the approval of fingolimod in 2010 [12, 13] was relatively simple because neurologists had two main treatment options: interferon beta/glatiramer acetate or natalizumab. The beta interferons and glatiramer acetate have comparable long-term safety profiles and efficacy, reducing the frequency of relapses by ~30% over a 2- to 3-year treatment period, as evaluated in clinical trials [1, 2, 14]. However, a high proportion of patients experience breakthrough disease or have persistent clinical or radiologic disease activity within 2 years of treatment initiation of these agents [14, 15]. Conversely, the safety of interferon beta and glatiramer acetate over two decades is highly favorable and the relative risk for immunologic complications is low [14, 16–18]. Natalizumab [19, 20] is more efficacious than interferon beta and glatiramer acetate [21, 22], but has a complex safety profile due to the risk of progressive multifocal leukoencephalopathy (PML) [19, 20]. For this reason, historically, natalizumab was generally reserved for patients requiring higher efficacy than interferon beta and glatiramer acetate, and, more recently in the US, only when the expected benefit is sufficient to offset PML risk [19, 20, 23].

Since 2010, new disease-modifying therapies (DMTs) have emerged [18]; 13 approved DMTs are currently available to treat RMS worldwide (Table 1), which target different pathways of the immune system (Table 2). Several of the new DMTs have demonstrated superior efficacy over either interferon beta or glatiramer acetate in Phase III studies of patients with RMS (Table 3). While there may be differences in their relative efficacy, additional head-to-head clinical trials, particularly comparator studies between oral DMTs, are required to confirm and quantify this assertion [24]. Oral DMTs such as teriflunomide, dimethyl fumarate, and fingolimod offer a convenient route of administration, while peginterferon beta, daclizumab beta, ocrelizumab, and alemtuzumab provide a lower dosing frequency, being dosed once every 2 weeks, monthly, every 6 months, and annually, respectively.

**Table 1:** Approved DMTs for MS

DMT (trade name)	Administration route	Dosing frequency	Dose level	US Approval year	Indication
IFN beta-1b (Betaseron^®^; Extavia^®^)	Subcutaneous injection	Every other day	250 mcg	1993^a^	RMS [134]
IFN beta-1a (Avonex^®^)	Intramuscular injection	Once a week	30 mcg	1996	RMS [135]
IFN beta-1a (Rebif^®^)	Subcutaneous injection	Three times per week	22 and 44 mcg	2002	RMS [136]
Peginterferon beta-1a (Plegridy^®^)	Subcutaneous injection	Once every 2 weeks	125 mcg	2014	RMS [137]
Glatiramer acetate (Copaxone^®^; Glatopa^®^)	Subcutaneous injection	Daily	20 mg	1996^b^	RMS [138]
		Three times per week	40 mg	2014	
Dimethyl fumarate (Tecfidera^®^)	Oral capsule	Twice a day	240 mg	2013	US: RMS [67]
					Europe: RRMS [139]
Teriflunomide (Aubagio^®^)	Oral tablets	Daily	14 and 7 mg	2012	US: RMS [72]
					Europe: RRMS [73]
Fingolimod (Gilenya^®^)	Oral capsule	Daily	0.5 mg	2010	US: RMS [13]
					Europe: second-line treatment or rapidly evolving severe RRMS [12]
Daclizumab beta (Zinbryta^®^)^d^	Subcutaneous injection	Once monthly	150 mg	2016	US: RMS, generally after an inadequate response to ≥2 DMTs^c^ [82]
					Europe: RMS and failure to respond/unsuitable for other treatments [81, 140]
Alemtuzumab (Lemtrada^®^)	Intravenous infusion	First course: daily for 5 days; second course: daily for 3 days, 1 year after the first course	12 mg	2014	US: generally reserved for patients who have had an inadequate response to ≥2 drugs for RMS^c^ [100]
					Europe: active RRMS defined by clinical or imaging features [141]
Natalizumab (Tysabri^®^)	Intravenous infusion	Every 4 weeks	300 mg	Approved 2004; reintroduced 2006	US: RMS when the expected benefit is sufficient to offset PML risk^c^ [20]
					Europe: high disease activity despite IFN beta or glatiramer acetate; rapidly evolving severe RRMS [19]
Ocrelizumab (Ocrevus^®^)	Intravenous infusion	Twice a year	600 mg	2017	US: RMS or PPMS [118]
Mitoxantrone (Novantrone^®^; Onkotrone^®^)	Intravenous infusion	Every 3 months	12 mg/m^2^	2000	US: secondary (chronic) progressive, progressive-relapsing, or worsening RMS (but not PPMS) as an early, transient, high-efficacy strategy [142]
		Every 3 months	5 mg/m^2^		

*DMT* disease-modifying therapy, *IFN* interferon, *MS* multiple sclerosis, *PML* progressive multifocal leukoencephalopathy, *PPMS* primary progressive MS, *RMS* relapsing forms of multiple sclerosis, *RRMS* relapsing–remitting multiple sclerosis

^a^Extavia available in the US since 2009

^b^Glatopa available in the US since 2015

^c^Only available through a restricted distribution program

^d^Formerly daclizumab high-yield process (approved as ZINBRYTA^®^), which has a different form and structure than an earlier form of daclizumab

**Table 2:** Mechanism of action and effects on the immune system of DMTs for RMS

DMT	Molecular mode of action	Effect on immune cells and mediators	Time taken for immune system reconstitution after DMT cessation
SC IFN beta-1b (Betaseron; Extavia) IM IFN beta-1a (Avonex) SC IFN beta-1a (Rebif) IM peginterferon beta-1a (Plegridy)	Exert autocrine and paracrine actions via activation of the IFN receptor on leucocytes	Reduces inflammatory cell migration across the blood–brain barrier, reduces the production of proinflammatory cytokines, and induces anti-inflammatory cytokines [18]	Effects on the immune system endure for five times the serum elimination half-life (i.e., 40 min to 21.5 h for SC IFN beta-1b [134], 95 h for IM IFN beta-1a [135], 345 h for SC IFN beta-1a [136], and 390 h for SC peginterferon beta-1a) [137]
Glatiramer acetate (Copaxone)	MBP mimetic; thus competes with MBP antigens to bind with MHC II [58]	Protects neurons by diverting T cell responses away from myelin in a dose-dependent manner. Increases production of anti-inflammatory cytokines and reduces the production of proinflammatory cytokines [18]	Effects on the immune system endure for five times the elimination half-life [138]
Dimethyl fumarate (Tecfidera)	Activates the nuclear factor (erythroid-derived 2)-like 2 pathway to protect against oxidative stress–induced cellular injury and loss in neurons and astrocytes [61]	Mean absolute lymphocyte count decreased by 30% during the first year then remained stable above the lower limit of normal (i.e., 0.91 × 10^9^/L) [63] 6% of patients had lymphocyte counts <0.5 × 10^9^/L [67] Effective immune response to recall antigens, neoantigens, and T cell-independent antigens similarly to nonpegylated IFN therapy [143]	>4 weeks for lymphocyte counts to increase, but did not return to baseline values [67]
Teriflunomide (Aubagio)	Inhibits proliferation of activated T and B lymphocytes via mitochondrial dihydroorotate dehydrogenase inhibition [144]	Reduces neutrophils and lymphocytes by 15%, with mean counts remaining in the normal range [71] 16% and 12% of patients had neutrophil and lymphocyte counts <1.5 × 10^9^ and <0.8 × 10^9^/L with 14 mg dose, respectively [72] Effective immune response to H1N1, H3N2, and B influenza strains [145]	Unknown; reduced white blood cell counts of 15% may be related to bone marrow suppression [72]
Fingolimod (Gilenya)	Binds the sphingosine-1-phospate receptor, blocking lymphocyte egress from lymph nodes [146, 147]	Dose-dependent reduction in peripheral lymphocyte count to 20–30% of baseline values [13] Lymphopenia incidence was 7% in placebo-controlled trials [13] Attenuated response to influenza vaccine in some patients [148]	≤2 months to return to normal range [13]; average lymphocyte counts were 80% of baseline values after 3 months [149]
Daclizumab beta (Zinbryta)^a^	Humanized monoclonal antibody that selectively blocks high-affinity IL-2 receptor formation on activated T cells. Modulation of the IL-2 signal leads to selective antagonism of activated T cell responses and expansion of immunoregulatory CD56^bright^ NK cells [81, 87]	Fivefold expansion in CD56^bright^ NK cells at 1 year. Total lymphocyte, CD4^+^ and CD8^+^ T cell, and B cell counts decrease ≤10% from baseline during the first year of treatment [81, 82, 92] Effective immune responses to influenza vaccine [150]	Pharmacodynamics are related to the half-life of daclizumab beta (21 days) and are reversible [55, 81, 82] Total lymphocyte counts return to baseline levels ~8–12 weeks after the last dose [81, 82] Treg and CD56^bright^ NK cell numbers return to baseline levels within 24 weeks [81, 93, 95]
Alemtuzumab (Lemtrada)	Targets CD52 on lymphocytes and monocytes. It readily depletes B cells, T cells, monocytes, macrophages, and dendritic cells, leading to long-lasting changes in adaptive immunity, and reduces the pathogenesis of inflammatory response in MS [99]	Decrease in the level of circulating T and B lymphocytes very rapidly, with the lowest values observed within days posttreatment [99]	Lymphocytes repopulate within 8 months, but T cell populations take >1 year to fully repopulate [100, 101] T cell populations do not recover to baseline levels [101]
Natalizumab (Tysabri)	Monoclonal antibody that selectively inhibits VLA-4 (α4β1) integrins, preventing leukocyte migration across the BBB [58]	Increases the number of circulating leukocytes (including lymphocytes, monocytes, basophils, and eosinophils) [20]. Natalizumab does not affect the absolute count of circulating neutrophils [20] Effective immune responses to recall antigen (tetanus toxoid) and neoantigen (keyhole limpet hemocyanin) [151]	≤16 weeks to return to baseline levels [20]
Ocrelizumab (Ocrevus)	Targets CD20 on B cells through mechanisms that include antibody-dependent cell-mediated cytotoxicity, complement-dependent cytotoxicity, and/or the induction of apoptosis [118, 152]	CD20^+^ targeted by ocrelizumab include pre-B cells, mature B cells, and memory B cells; lymphoid stem cells and plasma cells are unaffected [119, 152]	Median (range) time to B cell repletion to either baseline or lower limit of normal was 72 weeks (range 27–175 weeks); within 2.5 years after last infusion, B cell counts rose to either baseline or the lower limit of normal in 90% of patients [118]
Mitoxantrone (Novantrone)	Intercalates with DNA, causing strand breaks, and inhibits DNA repair via inhibition of topoisomerase II, leading to cytotoxicity [153]	Reduction of leukocytes primarily affecting neutrophils and most lymphocyte subsets except for naive and activated T lymphocytes [154]	Unknown

*BBB* blood–brain barrier, *DMT* disease-modifying therapy, *IFN* interferon, *IL*-*2* interleukin 2, *IM*, intramuscular, *MBP* myelin basic protein, *MHC II* class II major histocompatibility complex, *MS* multiple sclerosis, *NK* natural killer, *RMS* relapsing forms of multiple sclerosis, *SC*, subcutaneous, *Treg* regulatory T cell, *VLA*-*4* very late activation antigen 4

^a^Formerly daclizumab high-yield process (approved as ZINBRYTA^®^), which has a different form and structure than an earlier form of daclizumab

**Table 3:** Efficacy of DMTs for MS

DMT (trade name)	ARR reduction	Disability progression^a^	Neuroradiologic outcomes
IFN beta-1b (Betaseron; Extavia) 0.25 mg dose [155]	34%	No effect	0.9% reduction in lesion area from baseline versus a 21.4% increase with placebo [134]
IFN beta-1a (Avonex) [156]	18% for ITT population; 32% for completer population	37% relative reduction in 24-week CDP by end of year 2 [135]	Reduction in number of Gd^+^ lesions (0.80 vs. 1.65) and volume of Gd^+^ lesions per patient (74.1 vs. 122.4 mm^3^)
IFN beta-1a (Rebif) [157]	29% (22 mcg); 32% (44 mcg)	23% (22 mcg) and 31% (44 mcg) relative reduction in ≥12-week CDP [158]	Median 1.2% (22 mcg) and 3.8% (44 mcg) decrease in T2 lesion burden versus a 10.9% increase with placebo. Reduced the number of T2 active lesions by 67% (22 mcg) and 78% (44 mcg)
Peginterferon beta-1a (Plegridy) [159]	36% at 1 year	38% relative reduction in 12-week CDP and 54% reduction in 24-week CDP at 1 year	67% reduction in new or newly enlarging T2 lesions; 86% reduction in Gd^+^ lesions; 53% reduction in new T1 hypointense lesions at 1 year
Glatiramer acetate (Copaxone; Glatopa) [160]	29%	No effect	54% reduction in new or newly enlarging T2 lesions; 41% reduction in new T1 hypointense lesions; 61% reduction in mean Gd^+^ lesions [161]
Dimethyl fumarate (Tecfidera) [162]	49%	29% reduction in 24-week CDP	83% reduction in Gd^+^ lesion activity; 78% reduction in new or newly enlarging T2 hyperintense lesions; 65% reduction in new nonenhancing T1 lesions
Teriflunomide (Aubagio) [163, 164]	32–36%	30–32% reduction in 12-week CDP	80% reduced risk of Gd^+^ lesions; 67% reduction in total lesion volume; 31% reduction in T1 lesion volume; 77% reduction in T2 lesion volume [163]
Fingolimod (Gilenya) [165–167]	48–54% [165, 166] 38–52% versus IFN beta-1a 30 mcg/week IM over 1 year [167]	Data equivocal: FREEDOMS: 30% reduction in 12-week CDP [165]; no statistically significant effect in FREEDOMS II [166] No statistically significant effect in 12-week CDP versus IFN beta-1a 30 mcg/week IM over 1 year [167]	FREEDOMS: fewer Gd^+^ lesions (0.2 vs. 1.1) and new or newly enlarging T2 hyperintense lesions (2.5 vs. 9.8) [165] Data confirmed in FREEDOMS II [166] Fewer Gd^+^ lesions (0.2 vs. 0.5) and new or newly enlarging T2 hyperintense lesions (1.7 vs. 2.6) versus IFN beta-1a 30 mcg/week IM over 1 year [167]
Daclizumab beta (Zinbryta) [92, 168]^b^	SELECT: 54% over 1 year [92] DECIDE: 45% versus IFN beta-1a 30 mcg/week IM over 144 weeks [168]	SELECT: 57% risk reduction in 12- and 76% reduction in 24-week CDP over 1 year [81, 92] DECIDE: 27% risk reduction in 24-week CDP versus IFN beta-1a 30 mcg/week IM over 144 weeks [168]	SELECT: over 1 year: 85% reduction in odds of new Gd^+^ lesions; 70% reduction in number of new or newly enlarging T2 hyperintense lesions [92] DECIDE: 75% reduction in odds of Gd^+^ lesion activity and 54% reduction in number of new or newly enlarging T2 hyperintense lesions over 96 weeks versus IFN beta-1a 30 mcg/week IM [168]
Alemtuzumab (Lemtrada)	Treatment-naive patients		
	CAMMS223: 69 and 66% in relapse risk over 3 and 5 years, respectively, versus IFN beta-1a 44 mcg SC three times per week [37, 169] CARE-MS I: 55% in relapse risk over 2 years versus IFN beta-1a 44 mcg SC three times per week [170]	CAMMS223: 75 and 69% risk reduction over 3 and 5 years, respectively, versus IFN beta-1a 44 mcg SC three times per week [37, 169] CARE-MS I: No statistically significant effect over 2 years versus IFN beta-1a 44 mcg SC three times per week [170]	Improvement in lesion load on T2-weighted MRI, and cerebral volume on T1-weighted MRI in CAMMS223 [169] CARE-MS I: reduced the proportions of patients with Gd^+^ lesions (7% alemtuzumab vs. 19% IFN beta-1a 44 mcg SC three times per week) and new or newly enlarging T2 hyperintense lesions (48 vs. 58%)
	Treatment-experienced patients		
	CARE-MS II: 49% in relapse risk versus IFN beta-1a 44 mcg SC three times per week [105]	CARE-MS II: 42% reduction in 6-month risk versus IFN beta-1a 44 mcg SC three times per week	No between-group difference on T2 lesion volume; reduced the proportions of patients with Gd^+^ lesions (9% alemtuzumab vs. 23% IFN beta-1a 44 mcg SC three times per week) and new or newly enlarging T2 hyperintense lesions (46 vs. 68%)
Natalizumab (Tysabri) [171]	AFFIRM: 68%	AFFIRM: 42% risk reduction in 12-week CDP; 54% risk reduction in 24-week CDP	AFFIRM: 92% fewer Gd^+^ lesions and 83% reduction in new or newly enlarging T2 hyperintense lesions
Ocrelizumab (Ocrevus) [120]	OPERA I: 46% versus IFN beta-1a OPERA II: 47% versus IFN beta-1a	OPERA I and II: 40% risk reduction in 12- and 24-week CDP versus IFN beta-1a	OPERA I: 94% reduction in Gd^+^ lesions; 77% reduction in new or newly enlarging T2 hyperintense lesions versus IFN beta-1a OPERA II: 95% reduction in Gd^+^ lesions; 83% reduction in new or newly enlarging T2 hyperintense lesions versus IFN beta-1a
Mitoxantrone (Novantrone) [172, 173]	63% of relapse risk [172]	70% reduction in 24-week CDP [173]	Gd^+^ lesions (0 vs. 16% on placebo); mean increase in T2 lesions: 0.29 in the mitoxantrone group and 1.94 in the placebo group [172]

Efficacy data are over 2 years and relative to placebo unless otherwise stated

*ARR* annualized relapse rate, *CDP* confirmed disability progression, *DMT* disease-modifying therapy, *Gd* ^+^ gadolinium-enhancing, *IFN* interferon, *IM* intramuscular,* ITT* intention-to-treat, *MRI* magnetic resonance imaging, *MS* multiple sclerosis, *SC* subcutaneous

^a^Disability progression data are significant unless stated otherwise

^b^Formerly daclizumab high-yield process (approved as ZINBRYTA^®^), which has a different form and structure than an earlier form of daclizumab

Although the expansion in treatment options for RMS is welcome, health care professionals are now faced with complicated decisions on how to individualize initial therapy for patients (see Table 4 for prognostic features in MS) and then select subsequent therapies, based on incomplete benefit–risk assessments of the current and potentially unknown long-term immunologic and safety risks. The most important knowledge deficit is the long-term safety of newly approved DMTs for RMS, which may not have been fully elucidated during their Phase III clinical trial programs, and thus may place some patients at risk for complications yet to be defined. For instance, some DMTs for RMS have been associated with adverse events (AEs) that only came to light during postmarketing surveillance [16, 25], culminating in the development of intensive risk reduction strategies to optimize patient safety such as classifications of PML risk [26]. Other generic factors preventing the extrapolation of data to a real-world setting include strict patient selection and high motivation in clinical trials.

**Table 4:** Prognostic features in MS [2, 30, 39, 174–186]

Better prognosis	Poorer prognosis
White	African American or nonwhite
Female	Male
Younger age	Older age
Monofocal onset	Multifocal onset
Minimal cortical pathology	Early cortical involvement
Onset with optic neuritis or isolated sensory symptoms	Onset with motor, cerebellar, or bladder/bowel symptoms
Low relapse rate first 2–5 years	High relapse rate first 2–5 years
High degree of remission after first relapse	Short interattack latency
Long interval to second relapse	Short interval to second relapse
Mild relapse	Severe relapse ≥1 moderate or severe attack Steroids/hospitalization required Severe effect on activities of daily living >1 functional system affected Severe motor/cerebellar brainstem involvement
No or low disability at 5 years	Disability at 2 or 5 years
Low lesion load on MRI	Abnormal MRI ≥2 Gd^+^/new or newly enlarging T2 hyperintense lesions or ≥ 2 T1 hypointense lesions ≥2 spinal cord lesions Baseline brain atrophy
NEDA at 2 years	Disease activity at 2 years
Early treatment	Late treatment
Low (≤386 ng/L) neurofilament light levels	Elevated (>386 ng/L) neurofilament light levels
Absence of oligoclonal IgG bands	Presence of oligoclonal IgG bands and ≥10 brain T2 lesions
Absence of IgM bands	Presence of IgM bands

*Gd* ^+^ gadolinium-enhancing, *Ig* immunoglobulin, *MRI* magnetic resonance imaging, *MS* multiple sclerosis, *NEDA* no evidence of disease activity

The purpose of this review is to raise awareness of the issues involved in sequencing RMS therapies by discussing the immunologic effects and known safety profiles of available DMTs. In doing so, the treating neurologist may be better able to inform patients on the likely benefits and risks of treatment.

## General principles of treatment sequencing in RMS

The primary aim of treatment is to reduce disease activity to optimize neurologic reserve, cognition, and physical function [27]. Meeting this goal requires a concordant relationship between the health care professional and patient so that the personal preferences of the patient are considered when developing or revising the treatment plan (shared decision making). Patients should be informed that different DMTs may be required at different times because of suboptimal response, safety concerns, intolerable side effects to the DMT, or a change in the risk tolerance of the patient. Within this context, patients could be made aware of commonly used criteria necessitating a treatment switch due to suboptimal response. For interferon beta recipients specifically, the Rio score estimated after 1 year of treatment is prognostic for ongoing disease activity in the ensuing 3 years [28, 29]. For DMTs more generally, the Canadian Multiple Sclerosis Working Group recommends changing treatment when there is a low level of concern in all three domains of the MS disease status triad [relapses, disability progression, and magnetic resonance imaging (MRI)], a medium level of concern in any two domains, or a high level of concern in any one domain [30]. Another example is the multifactorial MS decision model that, by grading the four domains of relapse, disability progression, MRI, and neuropsychology, aims to support early treatment decisions and detect treatment failure in a timely manner [31].

Regardless of the clinical criteria used to identify suboptimally controlled RMS [30, 32], treatment sequencing is often necessary to maintain disease control, which may introduce an additional safety risk [30]. Neurologists have two major decisions regarding the prescription of DMTs: (1) choosing the initial DMT expected to reduce disease activity while recognizing the potential need for alternative later-line DMTs if the response is suboptimal; and (2) selection of subsequent treatment choices based on previous DMT use. The appropriate course of action selected depends on a thorough benefit–risk evaluation for each candidate DMT after accounting for specific disease- and patient-related factors at a specific point in time, as well as the patient’s access to DMTs through their health insurance plans.

Treatment algorithms for RMS that rank DMTs as first and second line have been proposed [33, 34], as have the pros and cons of induction (starting highly effective therapy earlier in the course of the disease) versus escalation treatment paradigms [35, 36]. However, the initial DMT should provide the most favorable benefit–risk profile given the level of disease activity over the last 6–12 months, taking into account the MS type, long-term prognostic factors (Table 4), patient-related factors, and the fact that the risk of AEs associated with some DMTs can change over time. Importantly, due to the heterogeneity of RMS, the choice of starting DMT should take into consideration potential future treatment needs by keeping subsequent treatment options open (e.g., reserving the need for DMTs with a long-lasting immunoablative impact on the immune system until a later time if appropriate). Like a good chess player who thinks several moves ahead, performing multiple benefit–risk assessments across several DMTs for RMS is required when reviewing medication at any point in time.

## Escalation versus induction

Clinicians may deliberate on the relative value of an escalation or induction treatment approach. A treatment escalation approach is based on starting with a relatively safer agent and reactive treatment switches due to breakthrough disease. At every stage in the patient’s disease course, there can be lasting effects of previous DMTs on the patient’s immune system, especially with medications that have prolonged immunologic sequelae. When switching from the initial DMT, it is imperative to consider the mechanism of action and duration of pharmacodynamic and immune system effects because these can impact the efficacy and safety of the next agent. Patients with moderate disease activity [one disabling relapse in the last year and/or two new gadolinium-enhancing (Gd^+^) lesions, or accumulation of two new T2 lesions per year, indicating multifocal attacks] or high disease activity (at least one disabling relapse in 1 year plus at least three new Gd^+^ lesions, or accumulation of three new T2 lesions per year) may be placed on a high-efficacy treatment early in the disease course and continue with that DMT [24, 30]. Some therapies with reversible mechanisms of action facilitate escalation of therapy towards other agents within a relatively short time frame of discontinuation if safety considerations allow, whereas other DMTs with long-term effects on the immune system after treatment cessation can limit the scope of subsequent pharmacotherapy. The latter DMTs also have been used as induction therapies.

Induction involves short-term use of a high-efficacy treatment to obtain rapid control of highly active disease and to increase the likelihood of beneficial long-term outcomes [35, 37], justifying an increased risk of serious AEs. The induction strategy is generally intended for younger patients (<40 years of age) with aggressive RMS who may have already received immunomodulatory drugs, with frequent (at least two) and severe relapses within the last 12 months, neuroradiologic activity (at least two additional Gd^+^ lesions on recent T2 MRI), and who are at increased risk of rapid accumulation of disability (e.g., high relapse rate in the first 2–5 years and short first interattack interval) [33, 35, 38], and people of African descent [39]. Before an induction strategy is initiated, physicians must consider the appropriate maintenance DMT postinduction but, in practice, this is not always possible and data to guide postinduction choices are very limited.

The range of available induction therapies is fairly narrow, and includes mitoxantrone, alemtuzumab, and to a limited extent, hematopoietic stem cell transplantation (within a clinical trial setting). Off-label cyclophosphamide also has been investigated in patients with highly active disease [40]. There are positive neuroradiologic data for the brief use of immunosuppressive induction therapy with mitoxantrone before maintenance therapy with glatiramer acetate in patients with highly active RMS [41, 42]. However, the safety profile of the induction agent may preclude many patients from receiving this treatment strategy. Use of either immunoablative chemotherapy or immune-depleting antibodies followed by autologous hematopoietic stem cell transplantation has been successful in treating patients with MS [43–45]. The immune-depleting antibody alemtuzumab may be considered as an induction therapy because its effects on the immune system persist long after treatment cessation, enabling dosing on a one-off or annual basis [37]. In contrast, the high-efficacy DMTs fingolimod and natalizumab should not be considered as induction therapies because their rapidly reversible mechanisms of action predispose patients to a quick return of disease activity following treatment cessation [46–53]. For a similar reason, it is likely that daclizumab beta should be used as a maintenance high-efficacy DMT rather than as an induction therapy [54, 55].

## Clinical pharmacology, safety, and monitoring of DMTs

Most DMTs have a clear pharmacodynamic drug–drug interaction by virtue of their temporal effects on immune cell counts and functions (Table 2). Because different DMTs exert distinct immunologic effects that persist for variable periods of time after discontinuation, the immune system may not have fully recovered to its pretreatment baseline physiologic composition during transition from one DMT to another. The type and duration of effect of the previous DMT not only influences the selection of the subsequent DMT, but also the known and unknown risk of an AE with the later-line DMT. As currently understood, the armamentarium of approved and investigational agents for MS can be grouped into DMTs that exert near-term effects on the immune system [day-to-week timescale: interferon beta-1a and 1b, peginterferon beta-1a, glatiramer acetate, dimethyl fumarate, teriflunomide (if an accelerated elimination procedure with activated charcoal or cholestyramine is used)], mid-term immunologic effects (week-to-month timescale: fingolimod, natalizumab, and daclizumab beta), and long-term immunologic effects [month-to-year timescale: mitoxantrone, teriflunomide (if the rapid elimination procedure is not implemented), alemtuzumab, and ocrelizumab]. A more precise classification of the immunologic half-lives of DMTs will be possible once their effects on lymphocyte subpopulations are better understood. The safety profile and immunologic effects of interferon beta and glatiramer acetate are such that an immediate transition to another DMT is possible, providing there are no obvious abnormalities on clinical biochemistry.

Arguably, a transition period between stopping the existing DMT and initiating a new DMT may be unnecessary with some of the newer DMTs, but there is a fine balance between the duration of washout (if any) and risk of disease. For instance, the immunologic effect of alemtuzumab persists long after cessation of therapy and is unrelated to its biologic half-life, which may expose patients to immune-mediated risks when they are switched to subsequent DMTs. However, aggressive rebound disease activity can resume shortly after stopping one agent and initiating another, as has been observed with natalizumab and fingolimod [46–48, 51], suggesting that better outcomes may be seen with shorter washout. Another point for consideration is whether a previous drug could potentially nullify or attenuate the mode of action of later-line therapies: would the B and T cell-depleting action of alemtuzumab occur immediately after use of fingolimod if lymphocytes have not yet exited from secondary lymphoid tissue? Hence, the diverse interactions of DMTs with the immune system underscore their efficacy and safety profile, which, in turn, guides patient monitoring.

The increased efficacy and/or patient convenience associated with newer DMTs relative to interferon beta and glatiramer acetate must be balanced against known and unknown safety issues. DMTs have the potential to produce on- and/or off-target-based toxicities that manifest as unexpected serious AEs. Safety concerns for some therapies only became evident during extension studies and postmarketing surveillance studies, requiring ongoing changes to several DMT product labels [16]. It follows that a more accurate benefit–risk assessment is possible for a DMT scrutinized by postmarketing surveillance than for a DMT that has completed Phase III clinical development, but has yet to undergo safety evaluation in a large patient population over a protracted period of drug exposure. For instance, a prospective descriptive study of patients with MS between 1995 and 2006 found an increased malignancy risk with the sequencing of multiple immunomodulatory and immunosuppressant therapies, and also with the number of immunosuppressant courses [56].

### Beta interferons, peginterferon beta-1a, and glatiramer acetate

The mechanism(s) of action of the beta interferons are not yet fully established, although these therapies have been available since the 1990s. All beta interferons are known to exert autocrine and paracrine actions via activation of the interferon receptor on leukocytes (Table 2). Production of proinflammatory cytokines is reduced, and production of anti-inflammatory cytokines is induced [18]. Attachment of a polyethylene glycol side chain to the parent interferon beta-1a molecule yields peginterferon beta-1a, which, when administered subcutaneously, has a longer half-life, higher systemic exposure, and lower immunogenicity potential than intramuscular interferon beta-1a [57]. Glatiramer acetate, a synthetic polymer of four amino acids (l-glutamate, l-lysine, l-alanine, and l-tyrosine), is a mimetic for the MS autoantigen myelin basic protein (MBP), and thus competes with MBP antigens for binding to class II major histocompatibility complex (MHC II) [58]. With formation of the MBP antigen/MHC II complex impeded by glatiramer, helper T cells have less opportunity for activation and potential to destroy myelin [58]. In addition, glatiramer binding to MHC II inhibits the interaction of MHC II with CD4^+^ molecules located on the surface of helper T cells (Th1 and Th2). Consequently, there is reduced production of proinflammatory cytokines (interferon gamma) by Th1 cells, increased production of anti-inflammatory cytokines (interleukin 10) by Th2 cells (promoting a less inflammatory state), and induction of antigen-specific expansion of FOXP3^+^ regulatory T cells [58, 59].

The effects of beta interferons and glatiramer acetate on the immune system likely only endure for as long as a patient is exposed to therapeutic drug concentrations (i.e., less than five times the elimination half-life; Table 2). The safety profiles of the beta interferons and glatiramer acetate are well established (Table 5).

**Table 5:** Tolerability, safety, and monitoring issues of DMTs for MS

DMT	Common AEs	Safety issues	Contraindications
IFN beta-1b (Betaseron/Extavia) [134]	Injection site reaction, lymphopenia, flu-like symptoms, myalgia, leukopenia (asymptomatic), neutropenia, increased liver enzymes, headache, hypertonia, pain, rash, insomnia, abdominal pain, asthenia, depression, hematologic abnormalities, arthralgia	Hepatic injury, anaphylaxis, depression (and suicidal ideation), injection site necrosis, congestive heart failure, leukopenia thrombotic microangiopathy, flu-like symptom complex, seizures, autoimmune disorders, decreased peripheral blood counts	Pregnancy (increased risk of spontaneous abortion) and breastfeeding Hypersensitivity reactions to active ingredient or formulation excipients
IFN beta-1a (Avonex) [135]			
IFN beta-1a (Rebif) [136]			
Peginterferon beta-1a (Plegridy) [137]			
Glatiramer acetate (Copaxone) [138]	Injection site reactions, postinjection reaction (vasodilatation, rash, dyspnea, chest pain within minutes)	Cutaneous necrosis	Use during pregnancy only if clearly needed Hypersensitivity reactions to active ingredient or formulation excipients
Dimethyl fumarate (Tecfidera) [67, 68]	Flushing, abdominal pain, diarrhea, nausea (usually subsides within 3 months) [187]	Anaphylaxis and angioedema, PML, lymphopenia	Hypersensitivity reactions to active ingredient or formulation excipients
Teriflunomide (Aubagio) [72–74]	Headache, diarrhea, nausea, alopecia, increased alanine aminotransferase	Hepatic injury, teratogenicity (requires accelerated elimination procedure), bone marrow effects, potential immunosuppression, infection, peripheral neuropathy, skin AEs [including Stevens–Johnson syndrome or toxic epidermal necrolysis (Lyell’s syndrome)], increased blood pressure, respiratory effects (interstitial lung disease), pancreatitis, thrombocytopenia	Severe hepatic impairment Pregnancy and breast feeding Severe immunodeficiency Significantly impaired bone marrow function Severe active infection Severe renal impairment undergoing dialysis Hypoproteinemia (due to high plasma protein binding) Current leflunomide treatment Hypersensitivity reactions to active ingredient, leflunomide, or formulation excipients
Fingolimod (Gilenya) [12, 13]	Headache, liver transaminase elevation, diarrhea, cough, influenza, sinusitis, infection, back pain, abdominal pain, pain in extremity	Asystole and sudden death, infections (including herpes simplex virus and cryptococcal infections), PML, macular edema, posterior reversible encephalopathy syndrome, respiratory effects, hepatic injury, teratogenicity, increased blood pressure, basal cell carcinoma	Recent myocardial infarction, unstable angina, stroke, transient ischemic attack, decompensated heart failure with hospitalization, or class III/IV heart failure History of Mobitz type II second- or third-degree AV block or sick sinus syndrome, unless patient has a pacemaker Baseline QTc interval ≥500 ms Known immunodeficiency syndrome Active infection Treatment with Class IA or Class III anti-arrhythmic treatment Active malignancy Severe liver impairment Hypersensitivity reactions to active ingredient or formulation excipients
Daclizumab-beta (Zinbryta) [81, 82]^a^	Nasopharyngitis, upper respiratory tract infection, rash, influenza, dermatitis, oropharyngeal pain, bronchitis, eczema and lymphadenopathy, depression, pharyngitis, increased alanine aminotransferase	Hepatic injury (including autoimmune hepatitis), other immune-mediated disorders (skin reactions, lymphadenopathy, noninfectious colitis), infections, and depression	Hypersensitivity reactions to active ingredient or formulation excipients Preexisting hepatic disease or hepatic impairment, including ALT or AST ≥2 times the ULN History of autoimmune hepatitis or other autoimmune condition involving the liver
Alemtuzumab (Lemtrada) [100, 141, 188–192]	Rash, headache, pyrexia, nasopharyngitis, nausea, vomiting, infection (urinary tract, upper respiratory tract, viral including herpes, fungal), fatigue, insomnia, urticaria, pruritus, thyroid gland disorders, arthralgia, pain in extremity, back pain, oropharyngeal pain, abdominal pain, diarrhea, sinusitis, paresthesia, dizziness, flushing	Infusion-associated reactions and anaphylaxis (including bradycardia), thyroid disorders and other autoimmune cytopenias, glomerulonephritis (Goodpasture’s disease), malignancy (thyroid cancer, melanoma, and lymphoproliferative disorders), infections (including opportunistic such as herpes virus, human papilloma virus, fungal infections, listeria, and nocardiosis)	HIV infection Hypersensitivity reactions to active ingredient or formulation excipients
Natalizumab (Tysabri) [19, 20, 193, 194]	Headache, fatigue, arthralgia, urinary tract infection, lower respiratory tract infection, gastroenteritis, vaginitis, depression, pain in extremity, abdominal discomfort, diarrhea, rash	PML, hypersensitivity reactions (including anaphylaxis), immunosuppression/infections (including herpes simplex virus, meningitis, and hepatitis B virus infection with acute fatal liver injury), hepatic injury	Patients who have or have had PML Patients at risk of opportunistic infections Concomitant beta IFN or glatiramer acetate therapy Known active malignancies (except cutaneous basal cell carcinoma) Hypersensitivity reactions to active ingredient or formulation excipients
Ocrelizumab (Ocrevus) [118]	Infusion-related reactions and upper respiratory tract infections	Infusion-related reactions, infections, neoplasms	Active hepatitis B virus infection and a history of life-threatening infusion reaction to ocrelizumab
Mitoxantrone (Novantrone) [142]	Nausea, alopecia, urinary tract infection, menstrual disorders (including amenorrhea), asthenia	Congestive heart failure (can result in death) may occur either during or months to years after termination of therapy. Secondary acute myeloid leukemia, infection, leukopenia, depression, bone pain, emesis, renal failure	Baseline LVEF below the lower limit of normal Pregnancy and breastfeeding Hypersensitivity reactions to active ingredient or formulation excipients

*AE* adverse event, *AV* atrioventricular, *DMT* disease-modifying therapy,* HIV* human immunodeficiency virus, *IFN* interferon, *LEVF* left ventricular ejection fraction, *MS* multiple sclerosis, *PML* progressive multifocal leukoencephalopathy,* ULN* upper limit of normal

^a^Formerly daclizumab high-yield process (approved as ZINBRYTA^®^), which has a different form and structure than an earlier form of daclizumab

### Dimethyl fumarate

Following oral administration, dimethyl fumarate is rapidly metabolized to its active metabolite monomethyl fumarate, which is primarily responsible for its efficacy in MS [60, 61]. Similar to other fumarate derivatives, administration of dimethyl fumarate activates nuclear factor-erythroid 2-related factor 2, resulting in differential effects involving antioxidant responses (Table 2) [60]. It is likely that dimethyl fumarate has several additional immunomodulatory effects underpinning its efficacy in MS, including directing the immune response away from Th1 [62].

Integrated analyses of patient-level data (*N* = 2470) from Phase IIb, Phase III, and long-term extension studies of dimethyl fumarate showed that mean absolute lymphocyte count decreased by 30%, but generally remained above the lower limit of normal during the first year of treatment, before stabilizing [63]. Separate observational data indicated that the dynamics of the absolute lymphocyte count generally correlate with CD4^+^ and CD8^+^ counts [64], with the reduction of CD8^+^ T cells greater than that of CD4^+^ T cells (−55 vs. −39%) reflected in a 36% increase in the CD4/CD8 ratio [65]. In patients without severe lymphopenia (i.e., <0.5 × 10^9^ cells/L), there is evidence of improvement in lymphocyte counts following discontinuation of dimethyl fumarate, but full restoration takes >4 weeks [63, 66, 67]. For patients who become severely lymphopenic on dimethyl fumarate, lymphocyte counts may take a long time to recover [66]; this delay in lymphocyte recovery may complicate the switch to a subsequent DMT with myelosuppressive effects. Six percent of patients experienced lymphocyte counts <0.5 × 10^9^/L (grade ≥3 lymphopenia) in placebo-controlled trials [67]. The risk of developing moderate to severe lymphopenia while on dimethyl fumarate may be increased by the following: increasing age, lower baseline absolute lymphocyte count, and recent natalizumab exposure (there is a greater percentage reduction in absolute lymphocyte count due to the lymphocytosis induced by prior natalizumab) [64, 66]. Although the risk may be slightly different in patients of older age or with lower baseline absolute lymphocyte count, all patients remain at a small risk of lymphopenia. Based on 7250 cumulative patient-years of exposure, the overall incidence of serious infections was low, and there was no apparent correlation between the incidence of infection and grade of lymphopenia [63].

Of >230,000 patients treated with dimethyl fumarate globally in the 3 years following commercial availability (representing >330,000 patient-years), there have been five cases of PML in the setting of moderate to severe prolonged lymphopenia (absolute lymphocyte count <0.8 × 10^9^/L), a rare opportunistic brain infection caused by John Cunningham (JC) virus that is normally harmless in immunocompetent hosts [25, 68]. There are established risk stratification and mitigation strategies for patients on dimethyl fumarate in light of its safety profile. In the US, complete blood counts at baseline and every 6 months thereafter are mandatory to identify patients who may have developed severe prolonged lymphopenia [67]. In Europe, a baseline MRI also should be performed within 3 months of initiating therapy [69].

### Teriflunomide

Teriflunomide is the active metabolite of leflunomide, which has anti-inflammatory, antiproliferative, and immunosuppressive properties [70]. Teriflunomide selectively and reversibly inhibits dihydroorotate dehydrogenase, a key mitochondrial enzyme in the de novo pyrimidine synthesis pathway, resulting in lymphocyte cell cycle impairment, without causing cell death [58]. Teriflunomide reduces neutrophils (during the first 6 weeks) and lymphocytes (during the first 3 months) by ~15%, although mean counts remain in the normal range [71]. Teriflunomide also inhibits protein tyrosine kinases, leading to decreased T cell proliferation, and a shift in the cytokine profile to a more anti-inflammatory cytokine milieu [58].

The long elimination half-life (18–19 days) of teriflunomide [72], due in part to its extensive enterohepatic recirculation, means that it can take approximately 8 months for the body to eliminate the drug, although plasma drug concentrations can still be detected up to 2 years after administration of the last dose [72]. If a rapid switch to another DMT is required, then an accelerated elimination procedure with either administration of cholestyramine or activated charcoal for 11 days should be considered [72], including in patients with cytopenia. Both elimination regimens result in a 98% decrease in plasma teriflunomide concentrations (i.e., <0.02 mg/L) [72, 73].

No new safety signals beyond those detected in individual trials (and summarized in Table 5) were identified with treatment duration exceeding 12 years and a cumulative exposure to teriflunomide exceeding 6800 patient-years [71]. Nonfebrile neutropenia and lymphopenia were reported in 5.9% and 0.5% of patients receiving teriflunomide over 1500 patient-years of cumulative treatment exposure, respectively [71]; no association between neutrophil count decrease and infection occurrence was detected [71]. Cases of thrombocytopenia, including rare cases with platelet counts <50,000/mm^3^, have been reported in the postmarketing setting [74]. Serious skin reactions, including severe generalized major skin AEs [Stevens–Johnson syndrome or toxic epidermal necrolysis (Lyell’s syndrome)] have been reported [74]. Teriflunomide is causally linked to one fatal case of toxic epidermal necrolysis [75], but has not been linked with PML. Complete blood count, tuberculin skin tests (to identify latent tuberculosis infection), liver function tests, and blood pressure measurements are required at baseline; liver function and blood pressure also should be monitored monthly for the first 6 months and then regularly thereafter with continued treatment [72].

### Fingolimod

Fingolimod is phosphorylated following oral administration to fingolimod phosphate, a mimetic for the naturally occurring extracellular lipid sphingosine-1-phosphate (S1P) [76]. S1P is an extracellular signaling molecule that regulates trafficking of many types of T and B cells from the lymph nodes to the blood (Table 2) [58]. Blood T cell levels decrease when S1P receptors are activated, as naive T cells are sequestered within secondary lymphoid organs after their egress from peripheral blood [58]. Lymphopenia is an expected pharmacodynamic effect of fingolimod due to the increased movement of CCR7^+^ lymphocytes into secondary lymphoid organs [77]. Fingolimod induces a rapid and reversible dose-dependent reduction in peripheral lymphocyte count to 20–30% of baseline values [13]. Peripheral lymphocyte reconstitution following fingolimod discontinuation occurs over 1–2 months [13], but this period may be extended with fingolimod use exceeding 1 year [78]. Rarely, an abrupt rise in lymphocyte count occurs during the fingolimod postwithdrawal period (immune reconstitution inflammatory syndrome), putting patients at risk for MS disease reactivation [79].

Cases of PML have occurred in patients with MS receiving fingolimod in the postmarketing setting who had not been previously treated with natalizumab nor who were taking immunosuppressive or immunomodulatory medications concomitantly [13]. In addition, affected patients had no other ongoing identified systemic medical conditions resulting in compromised immune system function [13]. For this reason, fingolimod should be withheld at the first sign or symptom suggestive of PML [13]. In addition, patients with signs and symptoms consistent with other opportunistic pathogens, including herpes simplex viruses 1 and 2, varicella zoster virus, cryptococci, and atypical mycobacteria, should undergo prompt diagnostic evaluation and appropriate treatment [13, 80].

Fingolimod phosphate acts on four of the five known S1P receptor subtypes expressed on a variety of cell types, including endothelial cells, lymphocytes, smooth muscle and cardiac myocytes, and neural cells [76]. Consequently, fingolimod administration elicits significant off-target pharmacology, resulting in rare but serious AEs (Table 5) [16]. For these reasons, in Europe, fingolimod is considered a second-line DMT following failure of interferon beta or glatiramer acetate, or a first-line agent for patients with highly active disease [12]. No such restrictions are placed on its use in the US, although numerous changes have been made to the warnings and precautions listed in the fingolimod product label to guide proper use (Table 5) [80]. Hence, patient selection and monitoring is of paramount importance to increase the likelihood that the benefits of fingolimod outweigh its risks. Baseline laboratory tests include complete blood count, liver enzymes, pregnancy test, and varicella zoster virus status [12, 13]. Tests that are required before dosing, during, and/or posttreatment with fingolimod include cardiac and blood pressure monitoring, complete blood counts, and examination of the fundus for macular edema [13]. Monitoring for signs of infection and suspicious skin lesions in case of basal cell carcinoma also should be conducted, as per the fingolimod product label [13]. Case reports of severe disease reactivation following fingolimod withdrawal [46–48] also must be considered when treating patients with highly active disease and in women who wish to become pregnant (in whom it is advised to continue therapy only if the potential benefit justifies the potential risk to the fetus) [12].

### Daclizumab beta

Daclizumab beta is a humanized monoclonal antibody approved for the treatment of RMS as a monthly subcutaneous self-injectable [81–83]. Daclizumab beta works in the periphery by binding CD25 (alpha subunit of the high-affinity interleukin 2 receptor mostly expressed on activated T cells) to modulate interleukin 2 signaling (Table 2) [84–87]. Blockade of CD25 by daclizumab beta limits interleukin 2 consumption by activated T cells and facilitates cells that express the intermediate-affinity interleukin 2 receptor [i.e., natural killer (NK) cells and precursors of innate lymphoid cells] to receive more interleukin 2 signal, as this receptor does not feature CD25 [88, 89]. Consequently, there is a substantial expansion of immunoregulatory CD56^bright^ NK cells that penetrate the blood–brain barrier and eliminate important mediators of MS immunopathology, activated T cells, leaving resting T cells intact (Table 2) [84, 86, 90, 91].

The pharmacodynamic effects of daclizumab beta are sustained in patients with relapsing–remitting MS, as evidenced by a fivefold expansion of CD56^bright^ NK cell levels that plateau by the end of the first year of treatment [92, 93]. Modest 10% reductions in circulating total lymphocytes, CD4^+^ and CD8^+^ T cells, and B cells were observed in patients with MS after 1 year of daclizumab beta 150 mg treatment, and regulatory T cell levels were reduced by approximately 50% after 8 weeks [92–94]. The remaining regulatory T cells are functionally active, as evidenced by stable cytokine production, maintained active cell cycling, and retention of a regulatory T cell-specific demethylated region in the FOXP3 promoter, albeit with a significant decrease in CD25 expression [94]. The effects of daclizumab beta are reversible; after treatment cessation, total lymphocyte counts return to baseline levels within 12 weeks, and CD56^bright^ NK cell and regulatory T cell counts return to baseline levels within 24 weeks [93, 95]. There was no apparent impact of antidrug antibodies or neutralizing antibodies on the pharmacodynamics, efficacy, or safety of daclizumab beta [82, 96].

The safety profile of daclizumab beta was determined over a 5-year period and was consistent with that from the pivotal Phase III trial (Table 5) [97]. Given the recent approvals for daclizumab beta, there are only limited data from real-world use, although the product label provides specific warnings regarding hepatic injury—elevations of serum transaminases and serious events, including fatal cases of autoimmune hepatitis and liver failure—and other immune-mediated disorders (including skin reactions, lymphadenopathy, and noninfectious colitis) as well as depression, infections, autoimmune hemolytic anemia, gastrointestinal AEs, and lymphopenia [81, 82]. These warnings are based on safety and tolerability information from an integrated analysis of six clinical studies (primarily randomized controlled trials and their extensions) encompassing 2236 patients with 5214 patient-years of daclizumab beta exposure [98]. Although this database was not large enough to detect rare events, the analysis did show that daclizumab beta had an acceptable safety profile without evidence of cumulative toxicity [98]. Daclizumab beta should be discontinued in cases of significant transaminase elevation [i.e., alanine transaminase (ALT) or aspartate transaminase (AST) >5 × the upper limit of normal (ULN) only; or total bilirubin greater >2 × ULN; or ALT or AST ≥3 but <5 × ULN and total bilirubin >1.5 and <2 × ULN] [82]. Discontinuing daclizumab beta should be considered if severe depression or suicidal ideation occurs [81, 82]. If serious infection develops, daclizumab beta should be withheld until the episode resolves [81, 82].

### Alemtuzumab

The humanized monoclonal antibody alemtuzumab targets the extracellular glycoprotein CD52, resulting in antibody-dependent cytolysis and complement-mediated lysis of T and B lymphocytes, monocytes, NK cells, macrophages, and dendritic cells (Table 2) [99, 100]. Alemtuzumab elicits rapid, profound, and prolonged B and T cell lymphopenia followed by a reconstituted immune system different in composition from that before treatment, which may rationalize its long-term efficacy, given patients only receive two medication cycles that are 1 year apart (Table 3) [99, 100]. It can take ~8 months for B cells and up to 3 years for T cell subsets to recover to the lower limits of the normal range after a single course of alemtuzumab, and T cells may not recover fully to baseline values [101]. It is worth noting that B cell recovery was rapid in one study; levels of ‘mature naive’ B cells (CD19 and CD23 positive but CD27 negative) returned to baseline by 3 months and rose to 165% of baseline values by 12 months after the first course of alemtuzumab treatment [102]. Conversely, CD27-positive memory B cell recovery was slow, reaching only 25% of baseline levels by month 12 [102]. The immunosuppressive effects of alemtuzumab on CD4^+^ T cell subsets lasted for up to 4 years in 29 patients who participated in CARE-MS I and CARE-MS II [103]. Differential lymphocyte reconstitution after alemtuzumab treatment may be a biomarker for MS relapse, as patients with active disease showed an accelerated recovery of CD4^+^ cells (*p* = 0.001), with a difference in absolute CD4^+^ counts at 24 months (*p* = 0.009), while CD4^+^ counts <388 × 10^6^ cells/mL predicted MRI stability [104]. Anti-alemtuzumab antibodies reduce plasma alemtuzumab concentrations during course 2 but not course 1, although they do not appear to affect clinical outcomes, total lymphocyte count, or AEs [100]. Alemtuzumab induces long-term immunodepleting effects, which must be considered when planning subsequent therapies for maintenance treatment or if a patient does not respond adequately. There are no data on sequencing therapies after alemtuzumab use to guide the clinician, who must, therefore, rely on intensive patient monitoring to individualize care.

Although the advantages of long-lasting efficacy and extremely high patient adherence are positive attributes of alemtuzumab, Table 5 shows that this DMT is associated with several serious AEs that may arise years after starting treatment and are, therefore, not reflective of alemtuzumab’s pharmacokinetic profile (elimination half-life, 2 weeks) [100]. Alemtuzumab is usually reserved for patients with unfavorable prognostic indicators because it is difficult to reconcile its superior efficacy over interferon beta with exposure to serious AEs in patients with less severe disease. Even in patients with highly active disease, diligent patient selection and strict adherence to risk monitoring programs is required. The alemtuzumab product label recommends regular laboratory monitoring up to 4 years after the last alemtuzumab dose (and beyond if warranted) for the detection of secondary autoimmune conditions (e.g., immune thrombocytopenia, antiglomerular basement membrane disease, and thyroid disorders) [100]. Laboratory testing includes differential blood count, serum creatinine, and urine analysis before administration and monthly thereafter [100]. Pretreatment thyroid-stimulating hormone level is mandatory and requires rechecking every 3 months until 4 years after the last infusion [100]. Patients with active or uncontrolled infections are not candidates for therapy [100]. Prophylactic oral acyclovir should be taken until CD4^+^ count is >200 cell/mm^3^ to reduce the risk for herpes infections [100, 105].

### Natalizumab

Natalizumab is a humanized monoclonal antibody that binds to the integrin molecule very late activation antigen 4 (α4β1), a glycoprotein surface molecule found on all leukocytes except neutrophils (Table 2) [58]. Blockade of α4β1 prevents adhesion of leukocytes to vascular cell adhesion molecule 1, a protein expressed on the surface of vascular endothelial cells in the brain and spinal cord, and thus blocks entry of leukocytes into the central nervous system across the blood–brain barrier [58]. Natalizumab increases the number of circulating leukocytes (due to inhibition of transmigration out of the vascular space), but does not affect the absolute count of circulating neutrophils [20].

Natalizumab has an elimination half-life of 11 days [20], although plasma natalizumab concentrations can be reduced by 92% within 1 week of plasma exchange sessions to treat PML if required [106]. The reversibility of natalizumab’s pharmacologic effects on peripheral immune cells is evident starting at weeks 8–12, with levels returning to those observed or expected in non-natalizumab patients by 16–20 weeks after the last natalizumab dose [107]. This is consistent with the reduction in plasma natalizumab concentrations to below the limit of detection by 16 weeks postdose [107]. Lymphocyte counts remain within the normal range at all times both for patients receiving natalizumab and for those who have stopped natalizumab treatment [107]. Patients who develop anti-natalizumab antibodies are more likely to have hypersensitivity reactions during drug administration [20].

An intensive risk stratification program is in place to help prescribers weigh the clear efficacy benefits of natalizumab against the development of PML [19, 20]. Three main factors drive the risk of developing PML in patients undergoing natalizumab therapy: (1) therapy ≥24 months; (2) previous use of immunosuppressant treatment; and (3) JC virus antibody positivity [108]. The anti-JC virus antibody index value and duration of natalizumab treatment are two key factors that enable clinicians and JC virus-positive immunosuppressant-naive patients with MS to make informed treatment and monitoring decisions [109–111].

Natalizumab withdrawal often leads to an MS relapse and return of inflammatory disease activity on MRI [49–53]. Younger patients (<40 years of age) were 3.8-fold more likely to have increased MRI activity during 24 weeks of natalizumab treatment interruption, as were those with one to five Gd^+^ lesions (2.7-fold increase) and >five Gd^+^ lesions (6.2-fold) before natalizumab initiation (vs. no lesions) [52]. Initiating interferon beta within 30 days postnatalizumab dosing in patients who had been free of disease activity [112], or initiating fingolimod ~4 months postnatalizumab dosing in patients with stable Expanded Disability Status Scale scores [113], was associated with clinical and radiologic disease recurrence. A therapeutic gap of no more than 3 months between discontinuing natalizumab and initiating fingolimod appears to minimize the risk of relapse [113]. Switching from natalizumab to alemtuzumab (*n* = 16) [114] or off-label rituximab (*n* = 114 [115] and *n* = 118 [116]) may be a feasible option to maintain disease control, including in those at high risk of PML. It is currently unknown if the switch-to therapy selection impacts the risk of PML in this context. Data on treatment selection after a PML event on natalizumab are limited, but there are reports of successful use of both dimethyl fumarate and fingolimod in this situation [117].

### Ocrelizumab

Ocrelizumab is a B cell-directed cytolytic monoclonal antibody with a humanized immunoglobulin G1 tail indicated for the treatment of patients with relapsing or primary progressive forms of MS as a twice-yearly intravenous infusion [118]. This recombinant monoclonal antibody binds to a different but overlapping CD20 epitope expressed on B cells to rituximab [119]. By binding to the cell surface antigen CD20 present on pre-B and mature B lymphocytes, it is believed that ocrelizumab induces antibody-dependent cellular cytolysis, complement-mediated lysis, and/or apoptosis via crosslinking membrane CD20 on the target cell surface [118, 119].

Assays for CD19^+^ B cells are used as a surrogate for B cell counts because ocrelizumab interferes with the CD20 assay. As such, ocrelizumab reduces circulating CD19^+^ B cell counts 14 days postinfusion to negligible levels [118]. In clinical studies, B cell counts rose to above the lower limit of normal or above baseline counts between infusions of ocrelizumab at least once in 0.3–4.1% of patients [118]. Median (range) time for B cell counts to return to either baseline or the lower limit of normal was 72 (27–175) weeks after the last ocrelizumab infusion (Table 2) [118]. Within 2.5 years after the last infusion, B cell counts rose to either baseline or the lower limit of normal in 90% of patients [118].

Treatment-emergent ocrelizumab AEs observed in a pooled analysis of the two identical 96-week Phase III OPERA trials included infections, infusion-related reactions, and an incidence rate of first neoplasm of 0.40 per 100 patient-years of exposure, based on data from 6467 patient-years of exposure (Table 5) [120]. The ocrelizumab prescribing information states that breast cancer occurred in 6 of 781 females treated with ocrelizumab versus none of 668 females treated with subcutaneous interferon beta-1a or placebo [118]. Hence, patients receiving ocrelizumab should be encouraged to follow standard breast cancer screening guidelines. The long-term effects and risks of B cell depletion on malignancy risk will remain uncertain until long-term real-world follow-up data are available, and may currently be underrecognized. As with alemtuzumab, long-term B cell depletion with ocrelizumab may limit subsequent treatment options. For instance, initiation of a later-line therapy while B cell levels remain depleted may result in cumulative, and presently undocumented, effects on immune system function. The appropriate timing for initiating other DMTs after a patient has received ocrelizumab should be considered by the physician. The immunogenicity of ocrelizumab appears low, based on the incidence of formation of treatment-emergent antidrug antibodies (~0.4%) [120].

## Balancing benefits versus risks: information for patients and physicians

Patient-related factors are key multifactorial inputs that influence response to DMTs, and how much a DMT is used in clinical practice. Selecting the treatment best suited for an individual at each phase of the disease is challenging, but can be facilitated by establishing a concordant relationship with the patient and their significant other (support partner). A key concept that may be disagreed upon in patients who feel relatively healthy is the value placed on future health benefits versus the present-day inconvenience of administering DMTs (e.g., tolerability, acquisition cost) and the safety profile of the DMT. Patient discussions provide an opportunity for the neurologist to relate the goals of the DMT to the patient, namely to safely reduce relapses and incidence and severity of new MRI lesions, thereby reducing the risk for permanent disability. Educating the patient about both the immediate symptomatic and long-term pathophysiological aspects of MS can facilitate the progression to a shared agreement about therapeutic goals and the level of risk patients and their partners are willing to assume. The neurologist can then help guide the patient regarding the long-term goals, general principles of sequencing DMTs, and the appropriate DMT treatment, rather than assessing their views and discussing details such as relative efficacy rates and disability rating scales.

There is some evidence of an effect on delaying disability progression with fingolimod, daclizumab beta, alemtuzumab, and natalizumab versus interferon beta or glatiramer acetate (Table 3). An appreciation of the treatment regimens from the patient’s perspective often reveals that their agendas and priorities may not match those of their neurologist, who should be guided by evidence-based medicine. The risk of the disease, which may be hard to precisely define, is another important part of the benefit–risk discussion in shared decision making. Hence, it is important to convey the advantages and disadvantages of each DMT to the patients based on their RMS history and likelihood/unpredictability of future disease-related events.

PML has been associated with several DMTs for MS, and one of the greatest needs in understanding the benefit–risk of a DMT is to quantify the likelihood of PML after the first DMT and also after two or more DMTs have been added in sequence. It is important to note that PML risk differs by therapy. A logical classification for stratifying DMT PML risk has been proposed, with natalizumab having the highest PML risk (incidence 1/100–1/1000), followed by far lesser degrees of risk for fingolimod (incidence 1/18,000) and dimethyl fumarate (incidence ~1/50,000) [26]. PML risk for other DMTs is very low or uncertain [26]. It is currently unknown whether or how the sequencing of these therapies might impact the overall PML risk of each patient.

The immunization status of the patient also is important because of interactions between some DMTs and vaccine response. The National Multiple Sclerosis Society does not recommend use of live vaccinations in people with MS [121], and respective product labels for teriflunomide, fingolimod, daclizumab beta, or alemtuzumab advise avoiding use of live attenuated vaccines during and for prespecified time periods after stopping therapy [13, 72, 82, 100]. No product-specific information is available on the effects of vaccination in patients receiving peginterferon beta-1a, glatiramer acetate, dimethyl fumarate, and natalizumab. Therefore, complete or partial prevention of infection by influenza, tuberculosis, varicella zoster virus, and hepatitis A and B may be considered by vaccination before starting immunomodulatory therapy. Availability of non-live vaccines, such as the herpes zoster subunit vaccine (HZ/su) containing recombinant varicella zoster virus glycoprotein E and the AS01_B_ adjuvant system [122], may represent a welcome and more flexible addition to efforts to prevent infection in patients with MS receiving DMTs.

For elderly patients, a potential benefit–risk consideration for the DMTs dimethyl fumarate, teriflunomide, fingolimod, alemtuzumab, and ocrelizumab is their variable effect on lymphocyte counts, which occur as a consequence of their on- or off-target pharmacology (Table 2). In elderly patients, age-related immunosenescence, characterized by diminished levels and functionalities of B and T lymphocytes, may lead to a theoretically greater likelihood of lymphopenia with these DMTs [123]. One also could hypothesize that elderly patients would be less likely to experience breakthrough MS activity for the same reason. Hence, older age affects the benefit–risk ratio of DMTs and acts as a prompt for neurologists to consider hematologic monitoring when making prescribing decisions.

Progress toward development of pharmacogenetics- and biomarker-based approaches to individualize treatments according to patient and DMT characteristics is in its infancy [124, 125]. In the meantime, other factors on which to base these decisions include patient preferences, lifestyle and beliefs, comorbidities and concomitant medications, immunization status, family planning, and age. The first three factors have a profound influence on adherence to medication; poor adherence predisposes the patient to suboptimal clinical, neuroradiologic, health-related quality of life, and pharmacoeconomic outcomes [126–131].

## Conclusions

The topics raised in our review also are emphasized in initial draft guidelines for the treatment of MS drawn up by the European Academy of Neurology, European Committee for Treatment and Research in Multiple Sclerosis, and American Academy of Neurology [132, 133]. Because both documents are highly data driven, there is no recommendation provided for selecting one DMT over another. Instead, the appropriate choice of DMT is the one that the practicing neurologist rationalizes will provide the level of efficacy warranted by the recent disease activity, balanced by patient safety and preferences.

The long-term immunologic and safety risks of sequencing multiple therapies are still unknown. Prescribing DMTs in RMS depends on a thorough benefit–risk analysis, which is inconclusive if the patient’s characteristics are not reflective of clinical trial populations, and if the long-term effects of DMT in the clinical practice setting are unknown. Prospective industry-sponsored switching studies, patient registries, and robust analysis of real-world data are needed to collect data tailored to the therapeutic agent and various patient scenarios. Until such evidence-based medical information is available, decisions on sequencing DMTs for RMS will depend heavily on the clinical acumen of the neurologist. In the meantime, sequencing the most appropriate therapies for patients with RMS is usually determined by a combination of factors such as disease activity, patient-related factors, and drug-related factors (e.g., pharmacodynamic profile).

Treatment should be selected to address the immediate clinical issue, and to keep alternative therapeutic options available for later-line therapies. This consideration is particularly important early on in the disease course and even more relevant in today’s therapeutic landscape, which includes DMT options with potentially long-lasting effects on the immune system that can persist for months or even years following discontinuation of therapy. Patients should be made aware of these issues so that a shared care decision can be reached, which is driven by matching the level of risk a patient is willing to accept with their prognostic factors.
